# Analysis of Adverse Events during Intrahospital Transportation of Critically Ill Patients

**DOI:** 10.1155/2017/6847124

**Published:** 2017-09-14

**Authors:** Francielli Mary Pereira Gimenez, Wesley Henrique Bueno de Camargo, Ana Clara Beraldo Gomes, Thaylla Sumyre Nihei, Monique Walicheki Maria Andrade, Maria Laura de A. F. Sé Valverde, Larissa D' Epiro de Souza Campos, Debora Carvalho Grion, Josiane Festti, Cintia Magalhães Carvalho Grion

**Affiliations:** ^1^Universidade Estadual de Londrina, Londrina, PR, Brazil; ^2^Hospital Evangélico de Londrina, Londrina, PR, Brazil; ^3^Universidade Federal Fluminense, Niterói, RJ, Brazil; ^4^Department of Internal Medicine, Universidade Estadual de Londrina, Londrina, PR, Brazil

## Abstract

**Purpose:**

To describe adverse events occurring during intrahospital transportation of adult patients hospitalized in an Intensive Care Unit (ICU) and to evaluate the association with morbidity and mortality.

**Method:**

Prospective cohort study from July 2014 to July 2015. Data collection comprised clinical data, prognostic scores, length of stay, and outcome at hospital discharge. Data was collected on transport and adverse events. Adverse events were classified according to the World Health Organization following the degree of damage. The level of significance was set at 5%.

**Results:**

A total of 293 patients were analyzed with follow-up of 143 patient transportations and records of 86 adverse events. Of these events, 44.1% were related to physiological alterations, 23.5% due to equipment failure, 19.7% due to team failure, and 12.7% due to delays. Half of the events were classified as moderate. The mean time of hospital stay of the group with adverse events was higher compared to patients without adverse events (31.4 versus 16.6 days, resp., *p* < 0.001).

**Conclusions:**

Physiological alterations were the most frequently encountered events, followed by equipment and team failures. The degree of damage associated with adverse events was classified as moderate and associated with an increase in the length of hospital stay.

## 1. Introduction

Intrahospital transport is defined as the temporary or definitive referral of patients within the hospital environment and may have a diagnostic and/or therapeutic purpose. It is a complex activity and must ensure the preservation of clinical conditions to those who are transported, throughout the course of the procedure [1, 2].

In practice, it is observed that transportation of hospitalized patients is often carried out automatically, without prior planning. This lack of planning may impair the preparation of the team, materials, and equipment and may facilitate the occurrence of adverse events [1, 3].

Studies have documented adverse events related to the following variables: multidisciplinary team, equipment, and physiological alterations inherent to the patient. Patient safety in the transportation scenario can be achieved with the use of appropriate equipment, trained staff, and the development of specific protocols [4, 5].

There are few studies evaluating the occurrence of adverse events during intrahospital transportation of severe patients and their association with significant clinical outcomes. In a cross-sectional study that analyzed 191 reports of adverse events over a six-year period, the authors reported team, patient management, and equipment failures [4]. More recently a multicenter study demonstrated a high incidence of adverse events occurring during the intrahospital transportation of critically ill patients, and variables related to the patient's clinical state before transportation were identified as risk factors for the occurrence of high risk adverse events [3].

This study aimed to describe adverse events occurring during intrahospital transportation of adult patients hospitalized in an Intensive Care Unit (ICU) and to evaluate the association with morbidity and mortality. The hypothesis of the study was that the occurrence of adverse events during intrahospital transportation of critically ill patients would be associated with increased morbidity and possibly increased mortality.

## 2. Methods

A prospective cohort study of patients admitted to an Intensive Care Unit (ICU) from July 2014 to July 2015.

The study was carried out in an adult Intensive Care Unit of a private philanthropic general hospital of high complexity, with 269 beds, being a reference for urgent and emergency care. The ICU studied is a tertiary urban center mixed unit (medical-surgical) of ten beds for the care of adult patients.

The sample consisted of all patients consecutively admitted to the ICU during the study period. Patients younger than 18 years, those in the ICU for less than 24 hours, and those who did not complete the signed informed consent form were excluded.

A data collection team, consisting of seven researchers, three nurses, and four medical students, was trained and prepared. Data collection was organized in a continuous and sequential way of patients admitted to the ICU during the study period. Data collection duty scales were prepared so that each week there was a member of the team responsible for the daily active search of the scheduled transports, in-person accompaniment of the transports, and a daily visit to the ICU to fill out the necessary forms. In cases of emergency transport, the ICU nurse contacted one of the researchers to accompany the transport.

Data collection was composed of demographic variables: initials of name, age, sex, and GR (general register) and clinical data: admission diagnosis, prognostic scores, presence of comorbidities, date of admission and discharge from hospital and ICU, and outcome at discharge from the hospital and ICU. On admission to the ICU, the* Sequential Organ Failure Assessment* (SOFA) and* Simplified Acute Physiology Score* (SAPS 3) scores were collected [6–9]. For the transported group, in addition to the cited data, the following data were collected: indication for transport, procedure to be performed, presence of invasive devices, place to be transported to, vital signs in the pre- and posttransport periods, continuous infusion pump (CIP), professionals involved, use of monitoring, transportation time, and occurrence of adverse events. The sources of the data were the records in the patient medical charts and real-time notes made during the transportation, which were transcribed for the instruments to carry out the research.

The indication for intrahospital transportation was made by the intensive care physician responsible for the patient. The reasons for intrahospital transportation were classified into two groups: intervention or diagnosis. Intrahospital transportation for diagnosis could include imaging examinations at the radiology and ultrasound center or at the hemodynamic laboratory and digestive endoscopy in the endoscopy room. Intrahospital transportation for therapeutic purposes could include surgical interventions in the surgical center, endovascular interventions in the hemodynamic laboratory, or endoscopic interventions in the endoscopy room.

Patients were first divided into two groups for comparison analysis: a group of patients requiring intrahospital transportation and a group of patients who did not require transportation. The prognostic indicators, in addition to the hospitalization time and outcome, were compared between these two groups. Subsequently the transported group was again divided into two further groups: a group of patients transported with the occurrence of adverse events and a group of patients transported without the occurrence of adverse events. The prognostic indicators, hospitalization time, and outcomes were compared between these two groups.

Comorbidities were defined according to the criteria published by the Charlson comorbidity index [10]. The procedures were categorized into procedure performed in the surgical center; procedure performed in the gastroenterology room; procedure performed in the hemodynamic laboratory; video examinations performed in specific sectors, and image examinations performed in the image sector.

Adverse events were defined as any event, expected or not, which influenced patient stability [11] and were divided according to the nature of the events into team failures; equipment failures; delays; and physiological alterations.

In order to classify adverse events, the International Classification of Patient Safety of the World Health Organization was used according to the degree of damage: None—no symptoms, or no symptoms detected and no treatment required, Mild—mild symptoms, loss of function or minimal or moderate damage, but with rapid duration, and only minimal interventions being required (e.g., extra observation, investigation, treatment review, and mild treatment), Moderate—symptomatic patient, requiring intervention (e.g., additional therapeutic procedure, additional treatment), with increased hospitalization time, permanent or long-term damage or loss of function, Severe—symptomatic patient, need for intervention for life support, or major clinical/surgical intervention, causing a decrease in life expectancy, with great damage or permanent or long-term loss of function, and Death—within the probabilities, in the short term that the event caused or accelerated death [11].

The data were analyzed in the program MedCalc Statistical Software version 15.2.2 (MedCalc Software Bvba, Ostend, Belgium). The level of significance adopted was 5% and the confidence interval 95%.

In the descriptive statistics, the continuous quantitative variables were described, after assessment of the adherence to normal distribution. For the variables with normal distribution, mean and standard deviation (SD) were calculated; otherwise, the median and interquartile ranges were calculated (percentile 25 and percentile 75). The nominal categorical variables were described as absolute and relative frequency (%).

In the analytical statistics, categorical variables were compared using Fisher's exact test. For the comparison of two groups of continuous variables with independent samples, the Student's *t*-test was used for variables with normal distribution. For cases where distribution was not normal, the Mann–Whitney test was applied. Correlations between two variables were measured by the Pearson coefficient.

This research was approved by the Research Ethics Committee Involving Human Beings of the State University of Londrina according to Opinion Number 036/2014, July 7, 2014, CAAE Number 26600914.1.0000.5231.

## 3. Results

**Figure 1:**
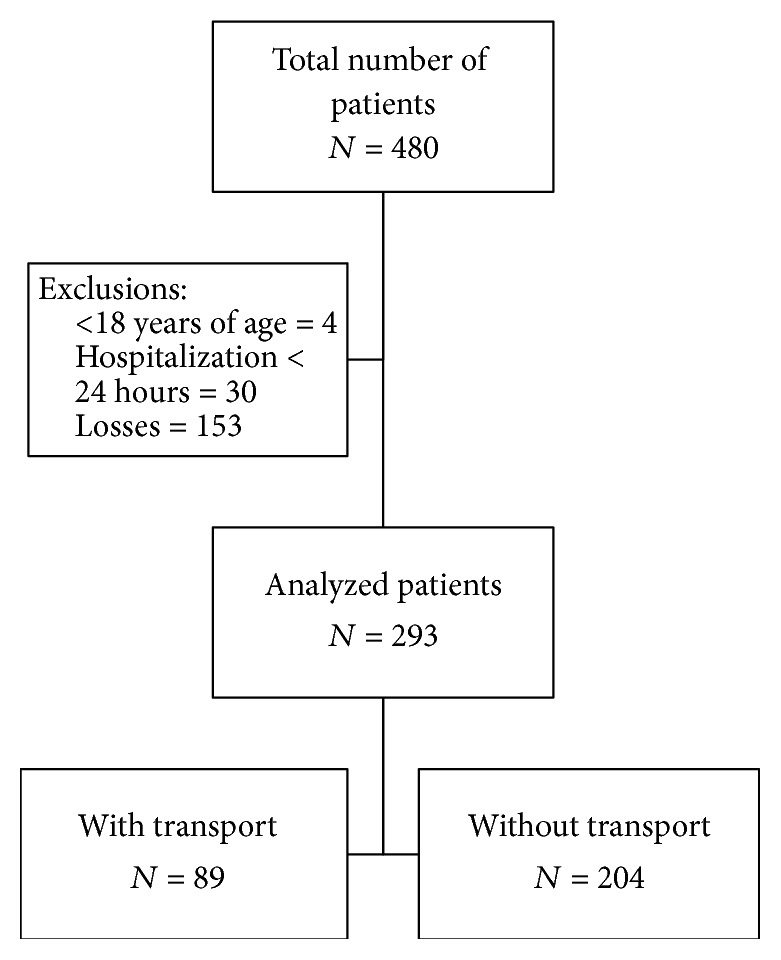
Flowchart of the study patients.

During the study period, 480 patients were admitted to the ICU and 187 patients were excluded, four of whom were under 18 years of age, 30 patients remained in the ICU for less than 24 hours, and 153 patients were considered as losses, since transportation occurred without the presence of a researcher for data collection. A total of 293 patients were analyzed during the period from July 2014 to July 2015. Of the patients studied, 89 were transported, totaling 143 transportations, since some patients required more than one transportation during hospitalization (Figure 1).

### 3.1. Characterization of the Study Population

Of the 293 patients included in the study, 53.9% were men and the median age was 66.5 (54.5–76) years. Regarding the sector of origin at the time of ICU admission, 35.8% of the patients were in the surgical center, 23.9% in the wards, 19.8% in the emergency department, 15% in the hemodynamic sector, 1.7% in another ICU, 1.4% in the intermediate care unit, 1.4% in other services, and 1% in the coronary unit.

Regarding ICU admission diagnoses, sepsis was the most frequent diagnosis (25.9%), followed by coronary insufficiency (15.4%), postoperative gastrointestinal complications (12%), postoperative admission due to chronic disease (8.2%), heart failure (4.4%), postoperative vascular surgery (4.4%), and other diagnoses (29.7%).

Comorbidities were present in 91.1% of the patients, the most frequent being hypertension (20%), diabetes mellitus (8.9%), chronic renal failure (6.6%), other endocrine diseases (5.8%), angina (5.1%), chronic obstructive pulmonary disease (4.5%), congestive heart failure (4.4%), other heart diseases (3.5%), and other comorbidities (41.2%).

### 3.2. Intrahospital Transports

Regarding the purpose, 57.3% of the transportations had a diagnostic purpose and 42.7% were therapeutic, 50.3% for imaging tests, 30.8% for central-surgical procedures, 15.4% for hemodynamic laboratory interventions, 2.1% for video exams, and 1.4% for procedures in the gastroenterology room.

All the transported patients presented one or more invasive devices, and the frequency of the devices for each patient was distributed as follows: 32.1% of the patients had one device, 19.6% had two devices, 24.5% had three devices, 10.5% had four devices, and 13.3% had five devices. The central venous catheter was the most frequent device (25.1%), followed by peripheral venous access (15.1%), delayed vesical catheter (14.9%), enteral catheter (13.5%), orotracheal tube (11.3%), and others (20.1%).

During the 143 transportations analyzed, on 74 occasions (51.7%) the patient was receiving one or more medications via continuous infusion pump. The medications used were fentanyl (19.1%), noradrenaline (17.8%), nitroglycerin (10.8%), hydrocortisone (9.6%), midazolam (5.1%), and other medications (37.6%).

On 17 occasions (11.9%) one professional was involved during the transportation, in 117 transportations (81.8%) two professionals were involved, and in 9 transportations (6.3%) there were three professionals. The professionals most frequently involved in transport were the nursing assistant, supervising nurse, and doctor.

The mean duration of transport time was 89 minutes (SD 91.51). The duration times ranged from 12 to 60 minutes in 81 (56.6%) transportations, from 61 to 120 minutes in 26 (18.2%) transportations, from 121 to 180 minutes in 20 (14%) transportations, from 181 to 240 minutes in 7 (4.9%) transportations, from 241 to 360 minutes in six (4.2%) transportations, and over 360 minutes in three (2.1%) transportations.

### 3.3. Adverse Events Related to Transportation

There were 86 adverse events that occurred in 57 of the 143 (39.9%) transportations performed. During a single transportation, more than one adverse event may have occurred.

Physiological alterations occurred in 44.1% of adverse events, with alterations in heart rate being the most frequent change. Equipment failure occurred in 23.5% of adverse events, with the oxygen tank finishing being the most common. Failure of the team occurred in 19.7% of the adverse events, interruption of manual ventilation or the continuous infusion pump being the most frequent. Delays occurred in 12.7% of the adverse events, attendance delay and obstacles present in the transit route being the most common.  Table 1 presents in detail the adverse events that composed the above groups.

For the classification of the degree of damage, 11.6% of the adverse events were associated with no damage, 38.4% of the events resulted in mild damage, and 50% of the adverse events were associated with moderate damage. There were no records of serious injury or death.

The mean time spent in the ICU was higher in the transported group of patients (14.8 days) than in the group of patients who did not require transportation (6.9 days, *p* < 0.001). The mean time of hospital stay was higher among patients who were transported (23.2 days) compared to patients who were not transported (17.2 days, *p* = 0.03) (Table 2).

Comparing transported patients with adverse events with those transported without adverse events, there was a tendency to an increase in hospital mortality among patients with adverse events (*p* = 0.07). The mean time spent in the ICU of the transported group who presented adverse events was higher compared to the patients transported without the occurrence of adverse events (21.7 days versus 9.2 days, resp., *p* < 0.001); in addition, the mean hospital time was also higher among patients with the occurrence of adverse events compared to those without adverse events (31.4 days versus 16.6 days, resp., *p* < 0.001) (Table 3).

The correlation between the number of transports per patient and the occurrence of adverse events resulted in a correlation coefficient of 0.40 (95% CI 0.21 to 0.56), *p* < 0.0001. The median transport time for patients without adverse events (60 minutes [33–180]) did not differ from the median transport time for patients with adverse events (60 minutes [32.5–120], *p* = 0.32). No difference was detected between the frequency of adverse events during therapeutic transportations (45.9%) compared to the frequency of adverse events during diagnostic transportations (35.2%, *p* = 0.22).

## 4. Discussion

From the results of the present study, we observed a high frequency of adverse events in the intrahospital transport of patients admitted to an ICU over a period of one year, resulting in increased morbidity in these critically ill patients.

In the present study, the incidence of adverse events related to physiological alterations was compatible with those found in the literature. Studies have shown that the most frequently encountered adverse events are physiological alterations which are detected in up to 79% of transported patients [3, 12–14].

The patients transported in this study were not monitored during transport, a reality that is not uncommon in hospitals in the geographic region where this study was carried out. The physiological alterations suffered by the patients during transport were only perceived when returning to the unit of origin. The same level of monitoring of physiological functions as at the unit of origin is recommended, including monitoring of the electrocardiogram, pulse oximetry, heart rate, respiratory rate, and continuous measurement of blood pressure throughout the transportation period so that appropriate measures can be initiated as soon as possible [13, 15–17].

Equipment failures occurred frequently in the present study, with incidents related to equipment being the most cited in the literature. The most common examples of equipment failures are the termination of the oxygen cylinders and the termination of equipment batteries [4, 13, 18, 19]. It is necessary to anticipate potential technical problems, in particular checking the oxygen cylinders and equipment batteries [20].

The most commonly reported team-related events are interteam communication failures; up to 61% of adverse events may be related to team failures [13]. Training and follow-up are suggestions for the professionals involved in this procedure to ensure all benefits and safety to the patient who is being transported [4, 12, 13].

In the present study some of the transportations suffered delays; these situations should be avoided, since they affect the staff, patient, and even the equipment batteries. It is recommended that the distance to be covered is examined, as well as possible obstacles, and communication be made with the destination sector at the time of transportation. If the place of destination is on a different floor, the lift should be in readiness [13, 21].

Adverse events were classified according to degree of damage. The majority received a moderate degree rating. This is perhaps the reason why there was no increase in the mortality of patients transported with adverse events, since a moderate degree is associated with an increase in hospitalization time, compatible with the results of the present study.

The majority of transportations in the present study had a diagnostic purpose. In the literature it is possible to find studies with a diagnostic purpose of up to 92.6% [13]. The implementation of bedside procedures contributes to a reduction in the risks of intrahospital transport; however, it is imperative that critically ill patients be discharged for complementary exams outside the ICU. Therefore, it is necessary to minimize the impact of risks to the patient [3, 13, 22].

The transport team could be considered as understaffed in a great proportion of the transportations analyzed in the present study. Studies recommend the presence of two to three trained professionals to accompany the critically ill patient: doctor, nurse, and physiotherapist in cases of mechanical ventilation [13, 15, 16, 23].

The increase in the mean length of hospital stay following the occurrence of an adverse event has already been described in the literature [24–26]. Longer periods of hospitalization may be required as a result of complications resulting from adverse events [27]. It is possible that better planning and training could reduce the occurrence of adverse events and contribute to a reduction in length of hospital stay and health costs.

The mean SOFA score was higher among the group of patients transported with the occurrence of adverse events than those transported without the occurrence of adverse events. The severity profile is a critical point to be observed in transportation planning. We found a weak correlation between the number of transportations per patient and the occurrence of adverse events. It would be logical to infer that the greater the number of transportations, the greater the chance of an adverse event; however it is possible that an adverse event has a greater association with the severity of the patient and the planning of the transport than with the number of occurrences. A recent study demonstrated that the patient's previous clinical condition is an independent risk factor for the occurrence of adverse events during intrahospital transport [3].

Our results do not demonstrate an association between the occurrence of adverse events and the duration of the transportation. There is divergence in the literature on this point, since some authors found a positive association between transportation time and the incidence of adverse events [28, 29], while others found an inverse association between the duration of transportation and the occurrence of adverse events [30]. Although we did not detect differences in the frequency of adverse events when we compared transportations with a therapeutic purpose and those with diagnostic purpose, there are reports that therapeutic interventions can generate physiological alterations and these alterations predispose the patient to complications during transportation [31]. It is possible that no increase was detected in the incidence of adverse transport events for therapeutic procedures due to the limited number of observations in the present study. Studies designed with statistical power to detect this difference may answer the question more appropriately.

The incidence of adverse events during intrahospital transportation may also be influenced by the type of institution and location. It is possible that, in rural or smaller hospitals, there are no patient monitoring protocols during or after intrahospital transportation, leading to underreporting of adverse events. The culture of safety of the hospitalized patient is being expanded globally; however it is possible that in low-income countries human and technological resources are limited and therefore adverse events are not routinely monitored or reported.

There are strengths and limitations to be considered in the present study. The limitations are due to the fact that a single center was studied and therefore may reflect a local experience, limiting its external validity. In view of the limited number of observations, it may not have been possible to detect small differences between the groups studied. The strength of the study is the methodological and data collection rigor through direct observation and the fact that this study evaluated the impact of the occurrence of adverse events on the patient's prognosis.

## 5. Conclusion

Adverse events that occurred in the intrahospital transportation of patients admitted to an ICU over the course of a year were described. Physiological alterations were the most frequently encountered events, followed by equipment and team failures. The degree of damage associated with the adverse events was classified as moderate in the majority of cases and was associated with an increase in the length of ICU and hospital stay.

## Figures and Tables

**Table 1:** Frequency of adverse events observed during intrahospital transport of critically ill patients.

Adverse events	*N*
*Physiological alterations*	38
Variation in HR ≥ 20 BPM	13
Hypertension	8
Hypotension	5
Variation in RR ≥ 10 RPM	4
Agitation	4
Saturation drop < 90%	1
Hypoglycemia	1
Bleeding	1
Vomiting	1

*Equipment failures*	20
End of O_2_ cylinder	15
End of the CIP battery	5

*Team failures*	17
Interrupted ventilation for 1 minute	3
Medication CIP interrupted	3
Loss of venous access	2
Returned from SC without medication forwarded	2
O_2_ tank accidentally closed	1
Patient without nasal catheter	1
Moved to the wrong location	1
Lack of communication between shifts	1
Medication error (wrong patient)	1
Secretion in the orotracheal tube	1
Taking medication for transportation without need	1

*Delays*	11
Delayed attendance	3
Obstacle on the transport path	3
Lift delay	2
Transported in bed not compatible with the lift	1
Team disagreement about medication	1
Door of the exam room locked	1

CIP: continuous infusion pump, SC: surgical center, O_2_: oxygen, HR: heart rate, BPM: beats per minute, RR: respiratory rate, RPM: breaths per minute, and ICU: Intensive Care Unit.

**Table 2:** Comparison of demographic data and prognostic scores of critically ill patients with and without intrahospital transport.

	With transport (*n* = 89)	Without transport (*n* = 204)	*p* value
Age^*∗*^	62.2/18.2	64.3/16.9	0.33^†^
Gender M	55%	53.4%	0.89^‡^
SOFA (day 1)^*∗*^	5/3.5	5.2/4.1	0.64^†^
SAPS^*∗*^	46.8/14.5	48.2/18.1	0.86^§^
Time in ICU^*∗*^	14.8/16.7	6.9/8.7	<0.001^§^
Time in hospital^*∗*^	23.2/22.6	17.2/22.3	0.03^†^
Mortality-ICU	28%	22.5%	0.37^‡^
Mortality-hospital	34.8%	28.9%	0.34^‡^

M: masculine, SOFA (day 1): *Sequential Organ Failure Assessment *at study entry, SAPS: *Simplified Acute Physiology Score*, and ICU: Intensive Care Unit; ^*∗*^mean/standard deviation; ^†^Student's *t*-test; ^‡^Fisher Exact; ^§^Mann–Whitney.

**Table 3:** Comparison of demographic data and prognostic scores of patients requiring intrahospital transport according to the occurrence or nonoccurrence of adverse events.

	With events (*n* = 40)	Without events (*n* = 49)	*p* value
Age^*∗*^	62.7/17.5	61.7/18.8	0.67^†^
Gender M	52.5%	57.1%	0.67^‡^
SOFA (day 1)^*∗*^	6.5/3.8	3.7/2.6	<0.001^§^
SAPS^*∗*^	49.9/17.6	44.4/11.1	0.10^§^
Time in ICU^*∗*^	21.7/21.3	9.2/8.4	<0.001^§^
Time in hospital^*∗*^	31.4/25.0	16.6/18.1	<0.001^§^
Mortality-ICU	35.0%	22.4%	0.23^‡^
Mortality-hospital	45.0%	26.5%	0.07^‡^

M: masculine, SOFA (day 1): *Sequential Organ Failure Assessment* at study entry, SAPS: *Simplified Acute Physiology Score*, ICU: Intensive Care Unit; ^*∗*^mean/standard deviation; ^†^Student's *t*-test; ^‡^Fisher Exact; ^§^Mann–Whitney.
